# A descriptive study of knowledge, beliefs and practices regarding osteoporosis among female medical school entrants in Sri Lanka

**DOI:** 10.1186/s12930-014-0015-y

**Published:** 2014-12-20

**Authors:** Risni Erandie Ediriweera de Silva, Muhamed Ruvaiz Haniffa, Kavinda Dimuthu Kumara Gunathillaka, Inoshi Atukorala, Endahandige Deepthi Prasadth Samantha Fernando, Wagawatta Liyanage Sugandhika Padmini Perera

**Affiliations:** Family Medicine Unit, Faculty of Medicine, University of Colombo, Kynsey Road, Colombo 8, Sri Lanka; Behavioural Sciences Stream, Faculty of Medicine, University of Colombo, Kynsey Road, Colombo 8, Sri Lanka; Department of Clinical Medicine, Faculty of Medicine, University of Colombo, Kynsey Road, Colombo 8, Sri Lanka; Department of Anatomy, Faculty of Medicine, University of Kelaniya, PO Box 6, Thalagolla Road, Ragama, Sri Lanka; Provincial Directorate of Health Services, R H Gunawardhana Mawatha, Badulla, Uva Province Sri Lanka

**Keywords:** Osteoporosis knowledge, Osteoporosis beliefs, Calcium intake, Osteo-protective behaviours, Womens health

## Abstract

**Background:**

Osteoporosis is a significant problem in rapidly ageing populations in Asian regions. It causes a significant personal and societal impact and increases the burden on health care services.

**Objectives:**

Aim of this study is to determine the knowledge, beliefs and practices regarding osteoporosis among young females entering medical schools in Sri Lanka.

**Methods:**

This is a descriptive cross sectional study conducted amongst 186 female medical school entrants of the Faculties of Medicine, Universities of Colombo and Kelaniya from September to December 2010. A self administered questionnaire was used to assess knowledge, beliefs and practices on osteoporosis, including a food frequency chart to assess the calcium intake.

**Results:**

The mean age was 20.7 +/− 2.1 years. Majority of the participants (51.6%, n = 96) had an average score (40–60) on the knowledge test, while 40.8% (n = 76) had a poor score (<40). However, in depth knowledge on risk factors, and protective factors was lacking. Perceived susceptibility for osteoporosis was low with only 13.9% (n = 26) of women agreeing that their chances of getting osteoporosis are high. The mean calcium intake was 528 mg/day and only 18.8% (n = 35) of the participants achieved the Recommended Daily Allowances (RDA) for Calcium. Exercise was grossly inadequate in the majority and only 13.6%( n = 23) engaged in the recommended exercises. Only 3.8% (n =7) of the participants currently engaged in specific behaviours to improve bone health while 10.8% (n = 20) had thought of routinely engaging in such behaviours.

**Conclusions:**

Although majority of participants had a modest level of knowledge on osteoporosis, there were gaps in their knowledge in relation to risk factors, protective factors and on the insidious nature of osteoporosis. Perceived susceptibility for osteoporosis was low. Practices towards preventing Osteoporosis were inadequate.

## Background

Osteoporosis is a global health problem. Osteoporosis and fractures secondary to osteoporosis are an important cause of mortality and morbidity [1]. Approximately 1.6 million hip fractures occur each year worldwide, the incidence is set to increase to 6.3 million by 2050 [1].

Due to increasing proportions of ageing populations in the Asian region, osteoporosis has become more prevalent and increases the health care expenditure in this region [2,3]. As osteoporosis is a silent disease, it is intuitive that primary preventive measures including early detection of risk factors for osteoporosis, educating the individuals at risk on preventive measures, and timely intervention with regard to treatment will reduce the morbidity and mortality and cut down the cost of treatment [4,5].

Females are at higher risk of developing osteoporosis. Female medical school entrants consist of an educated young population, with a gender-based higher risk of developing osteoporosis later in life. Studies carried out among females report that their knowledge about osteoporosis is limited [6-9]. A study carried out in Australia among Asian women revealed that their calcium intake was lower and their knowledge on osteoporosis was poor [10]. It is known that knowledge on osteoporosis contributes to enhance behaviours towards preventing osteoporosis [9].

Several factors have been recognized as critical towards improving bone health. These include modifiable factors such as calcium intake, physical activity, and non-modifiable factors such as genetic makeup [11,12]. Some of the established risk factors include ageing, female gender, family history of osteoporosis, early menopause, cigarette smoking, excessive alcohol consumption and certain medications [13]. It is reported that bone gain occurs in young adults till the age of 30 years [14]. Primarily, preventive strategies should be aimed at young women, as these interventions have the potential to delay the onset of osteoporosis. However, studies on the subject among young females in South Asian countries are scarce.

This study assesses the knowledge and beliefs on osteoporosis and practices towards preventing osteoporosis among young females of two major medical schools in the country.

## Methods

This study was carried out among the female medical school entrants of the Faculties of Medicine, at the Universities of Colombo and Kelaniya from September to December 2010. A pre-tested self administered questionnaire was used to assess knowledge, beliefs, and practices towards osteoporosis. Osteoporosis Knowledge Assessment Tool (OKAT), a validated tool was used [15]. This consists of 20 questions to assess knowledge. Content areas assessed include knowledge on osteoporosis risk factors, preventive measures, diagnosis, and management. The Osteoporosis Health Belief Scale was used to assess beliefs [16]. This scale includes questions on perceived susceptibility (3 questions), perceived barriers to Calcium intake (3 questions), perceived benefits of Calcium (3 questions), perceived seriousness (3 questions) and perceived health motivation (3 questions) [16].

The questionnaire to assess practices related to osteoporosis included questions on positive and negative behaviours towards osteoporosis. The positive behaviours assessed were dietary calcium intake, physical activity and exposure to sun. The negative behaviours assessed were on smoking status and alcohol intake. The questionnaire included a modified validated 40 item food frequency questionnaire to assess the calcium intake [17,18]. The food frequency questionnaire also included local calcium dense food items, after wide literature review and expert opinion.

Informed consent was obtained prior to the study. Ethical clearance for the study was obtained from the Ethics Review Committee of the Faculty of Medicine, University of Colombo.

### Data analysis

The variables were grouped in to blocks of knowledge, beliefs and practices, and within these blocks we have used descriptive statistics and the Pearson’s chi-square test to compare the two populations studied. The results were analysed using SPSS (version 19) and Excel computer software. A p value of <0.05 was considered as significant.

The knowledge on osteoporosis was scored as follows: each correct response scored one point and each incorrect response and “Do not know” response scored zero points. The total correct responses were given a score out of 20, and multiplied by five to obtain a score out of 100. The scores were categorized as follows- <20: Very Poor, 20–39: Poor, 40–59: Average, 60–79: Good and 80 or > 80: Very Good.

The questionnaire to assess practices was pre-coded to indicate positive and negative behaviours towards osteoporosis, and the individual behaviours were analysed separately. The calcium intake was calculated based on the data recorded in the 40 item food frequency questionnaire.

The number of portions of each food item consumed per week, as defined by the questionnaire was obtained for each participant. The amount of calcium contributed by each food item was derived by calculating the calcium content of food items taken from references. The weekly calcium intake for each respondent was calculated and the average daily calcium intake was obtained from this value. The total daily calcium intake was categorized as adequate (750 mg or more) and inadequate (less than 750 mg) based on the recommended daily allowance for calcium for young Sri Lankan females [19]. Physical activity was assessed based on the type and duration of weight bearing exercises engaged during a week.

## Results

The response rate was 100% with 86 participants from the University of Colombo and 100 participants from the University of Kelaniya. The mean age of the participants was 20.7 +/− 2.1 (mean +/− SD) years. The knowledge score on osteoporosis revealed that, 96(51.6%) had an Average score, while 71(38.2%) had a Poor score, and 5(2.7%) had a Very poor score based on the categorisation defined in the methodology. 14(7.5%) of the participants had a good score while none had a score above 80. The mean and median scores were 34.8 +/− 10 and 35 respectively, out of a total score out of 100.

Table 1 shows that, only 60(32.3%) recognized that a family history of osteoporosis is a risk factor for osteoporosis. Old age was not considered a risk factor by 81(43.5%), whilst 158(84.9%) did not know that smoking was a risk factor. 150(80.6%) did not know that a higher peak bone mass is protective. 111(59.7%) did not know that weight bearing exercises were protective for osteoporosis, however the students from the University of Kelaniya had better knowledge on the protective effect of weight bearing exercises on osteoporosis (p <0.01). The dietary sources of calcium were not known by 67(36%), and the students from University of Colombo demonstrated better knowledge for this question (p < 0.01). Only 34(18.3%) participants were aware that osteoporosis is usually asymptomatic.

**Table 1:** **Percentage of correct responses for osteoporosis risk factors and preventive practices**

**Risk factor/Preventive practice**				
	**Total (n = 186)**	**University of Colombo (n = 86)**	**University of Kelaniya (n = 100)**	P value*
Family history of osteoporosis	60 (32.3)	32 (37.2)	28 (28.0)	0.19
Female sex	177 (95.2)	81 (94.2)	96 (96.0)	0.56
Old age	105 (56.4)	54 (62.8)	51 (51.0)	0.11
Premature menopause	14 (7.5)	4 (4.7)	10 (10.0)	0.17
Smoking	28 (15)	17 (19.8)	11 (11.0)	0.10
Physical activity ( Weight bearing exercises)	75 (40.3)	25 (29.1)	50 (50.0)	< 0.01
Daily calcium requirement from food	84 (45.2)	41 (29.1)	43 (43.0)	0.52
Sources of calcium	119 (63.9)	70 (81.4)	49 (49.0)	<0.01
Hormone therapy after menopause	45 (24.2)	22 (25.6)	23 (23.0)	0.44

Data are presented as n (%).

*The Pearson’s Chi-square test was performed.

### Beliefs

Table 2 shows that, only 26(13.9%) women agreed that their chances of getting osteoporosis were high. Perceptions, towards barriers to calcium intake and benefits of calcium intake revealed that 55(29.5%) participants felt eating calcium rich food was difficult, while 14 (7.5%) felt that they are unable to tolerate calcium rich foods or that they dislike calcium rich foods, and 15(8%) felt that calcium rich foods are too expensive. On assessing the perceived seriousness of osteoporosis, 100(53.7%) felt that if they had osteoporosis that it would change their whole life. 102(54.8%) mentioned that the thought of osteoporosis scares them, and 155(83.3%) felt that having osteoporosis would make daily activities more difficult.

**Table 2:** **Affirmative responses regarding beliefs on Osteoporosis**

**Perceived Susceptibility**	**Total (n = 186)**	**University of Colombo (n = 86)**	**University of Kelaniya (n = 100)**	P value*
Chances of getting osteoporosis are high	26 (13.9)	15 (17.4)	11 (11.0)	0.20
We are more likely to get the disease	35 (18.8)	20 (23.3)	15 (15.0)	0.15
Family history makes us more likely to get osteoporosis	17 (9.1)	4 (4.7)	13 (13.0)	0.05
**Perceptions, towards barriers to calcium intake**				
Eating calcium rich food is difficult	55 (29.5)	28 (32.6)	27 (27.0)	0.21
Calcium rich foods do not agree with us or we dislike calcium rich foods	14 (7.5)	5 (5.8)	9 (9.0)	0.41
Calcium rich foods are too expensive	15 (8)	5 (5.8)	10 (10.0)	0.30
**Perceptions, towards benefits of calcium intake**				
Eating calcium rich foods reduces risks of broken bones	157 (84.4)	72 (83.7)	85 (85.0)	0.81
Eating calcium rich foods helps to build bones	131 (70.4)	57 (66.3)	74 (74.0)	0.25
Eating calcium rich foods prevents future problems from osteoporosis	153 (82.2)	71 (82.6)	82 (82.0)	0.92
**Perceived seriousness of osteoporosis**	**Total (n = 186)**	**University of Colombo (n = 86)**	**University of Kelaniya (n = 100)**	P value*
If we had osteoporosis it would change our whole life	100 (53.7)	50 (58.1)	50 (50.0)	0.27
Thought of osteoporosis scares us	102 (54.8)	50 (58.1)	52 (52.0)	0.40
Having osteoporosis would make daily activities more difficult	155 (83.3)	75 (87.2)	80 (80.0)	0.19
**Health motivation towards osteoporosis**				
We are motivated frequently do things to improve our health	116 (62.3)	42 (48.8)	74 (74.0)	<0.01
We are motivated to eat a well-balanced diet	110 (59.1)	41 (47.7)	69 (69.0)	<0.01
Motivated to exercise regularly	41 (22)	12 (14)	29 (29.0)	0.01

Data are presented as n (%).

*The Pearson’s Chi-square test was performed.

Health motivation towards osteoporosis as shown in Table 2, indicate that 116(62.3%) were motivated frequently to do changes to improve their health, and 110 (59.1%) were motivated to eat a well-balanced diet, however only 41(22%) were motivated to exercise regularly. When considering the two medical schools, students from the University of Kelaniya indicated better health motivation towards osteoporosis (p < 0.01). Health motivation towards osteoporosis was satisfactory; however motivation towards exercise was poor.

### Practices

The mean calcium intake was 528 mg/day and only 35(18.8%) of the participants achieved the Recommended Daily Allowances (RDA) for Calcium. When considering the respondents who met the RDA, 9(4.8%) were taking supplements in the form of multivitamins, while 26(14%) were not taking any calcium supplements. The top calcium providing food groups consumed by the participants amongst the daily intake of food types were, milk, tea with milk, yoghurt, small fish, rice, and cheese. As indicated in Table 3, only 23(13.6%) engaged in the recommended exercises in type and duration. Walking was found to be the commonest mode of exercise. 158(85.9%) were exposed to the sun for at least 30 minutes a week. 184(99%) of the participants were teetotallers and non-smokers. Only 7(3.8%) participants were currently engaged in specific behaviours to improve bone health whilst 20(10.8%) had thought of routinely engaging in such behaviour.

**Table 3:** **Duration of weight bearing exercise per week in the study population**

	**Total (n = 186)**	**University of Colombo (n = 86)**	**University of Kelaniya (n = 100)**	P value*
<30 min/Week	79 (46.7)	37 (43)	42 (42.0)	0.62
30 min – 60 min (1 hr)	42 (24.9)	11 (12.8)	31 (31.0)	<0.01
60 min – 90 min	25 (14.8)	13 (15.1)	12 (12.0)	0.53
>90 min	23 (13.6)	15 (17.4)	8 (8.0)	0.05

Recommended duration of weight bearing exercise per week: > 90 min.

Data are presented as n (%).

*The Pearson’s Chi-square test was performed.

## Discussion

The aim of the study was to assess knowledge, beliefs and practices regarding osteoporosis among female medical school entrants. Medical school entrants were selected for the study as they would not have been exposed to any teaching regarding osteoporosis in the medical school. It was inferred that these study subjects would possess only the knowledge of a high school biology graduate. In addition, this was a young group of females wherein interventions to improve bone health had the potential to be of the highest long-term value.

The basic demographic characteristics, such as age and the level of education were similar as all students in the country study the same curriculum and sit for the same advanced level examination and qualified to medical schools based on merit. Also all medical schools in the country receive medical students from all parts of the country. Although it can be hypothesised that these students are representative of female medical school entrants in Sri Lanka generally, we do not have the data to make a definitive pronouncement on this. Therefore this is a limitation of this study in terms of the applicability to the wider Sri Lankan medical student entrant.

Although it is perceived that medical school entrants are amongst the best students in the country and are expected to have a high level of knowledge even prior to entry to medical school the general knowledge on osteoporosis, among our participants was at a modest level, with only half of the population achieving an average score and a mere 7.5% achieving a good score based on the total knowledge score questionnaire. The mean and median scores were 34.8 +/− 10 and 35 respectively out of a total score out of 100. Similar results were seen in a study among Salvadorean women aged 25–35 years which revealed a mean score of 12.1 out of 42 points (percentage 28.8) [20], although it must be noted that this study was carried out among the general population.

Knowledge on risk factors and preventive practices are important in preventing or delaying the onset of Osteoporosis as well as in minimising morbidity due to Osteoporosis. However, the knowledge on osteoporosis risk factors among our study participants appeared to be poor compared to a similar study among college women in the US [14]. Another study among medical students from a medical school in Turkey revealed that knowledge on risk factors for osteoporosis, nutritional factors and diseases resulting in osteoporosis were poor [8]. It has been reported in several studies that women possess limited knowledge about the disease, and are not taking adequate measures to prevent or treat osteoporosis as they age [7,21].

The importance of health motivation in influencing health related behaviours is described by Kim et al. [16]. Perceived susceptibility towards osteoporosis appeared to be low in our study population, with only 13.9% of women agreeing that their chances of getting osteoporosis are high. A study in New Zealand among females aged 20–49 years showed that the perceived personal susceptibility was 29% [22] and Edmonds *et al.* also reported similar results [23]. More than half of the study population perceived osteoporosis as a serious disease. Von Hurst *et al.* revealed that there was higher level of agreement about the seriousness of osteoporosis, however only less than a quarter of the subjects regarded osteoporosis as a crippling disease [22]. Barriers towards calcium intake were low and perceived benefits of calcium intake were high, indicating that our study population was motivated towards taking calcium rich food.

Farr *et al.* reported that low levels of physical activity may compromise bone development in young girls [24]. It is also reported that physical activity and strength were positively associated with Bone Mineral Density (BMD) even in very elderly men [25]. In our study, exercise was grossly inadequate in the majority with a mere 13.6% engaged in the recommended exercises. When compared with a similar study among college women in the US, 59.8% were getting inadequate osteo-protective exercise [14]. Wallace *et al.* reported lean mass to be a powerful predictor of BMD in young women. They reiterated that lean mass can be modified to some extent by physical activity, thus physical activity must be increased throughout the lifespan [26].

Reduced calcium intake is linked to the osteoporosis risk in later life. Dietary calcium is important in achieving optimal peak bone mass early in life, and having increased bone mass in middle aged and elderly women [27]. Only 18.8% of the participants achieved the RDA for Calcium, despite being motivated towards taking calcium rich food. The mean calcium intake in our study population was 528 mg/day. In a study carried out among Chinese women aged 21–30 years, mean dietary calcium intake was 448 mg/day [28]. Those with a dietary calcium intake of at least 600 mg/day had a 4%–7% higher mean bone mineral density at the spine and femur when compared with those with a mean intake below 300 mg/day [28]. Nearly all Asian countries are reported to be far below the Food and Agriculture Organisation (FAO) and the World Health Organisation (WHO) recommendations for calcium intake [29]. Majority of the study population is not engaging in osteo-protective behaviours as evidenced by their poor consumption of calcium rich food and lack of physical activity. This is also reported in other studies [15,23], where the need for osteo-protective behaviours has been emphasised.

With respect to the results of this study, comparing the knowledge, beliefs and behaviours of the students of the two different medical schools significant differences in knowledge was not seen in most parameters. However, participants from the University of Kelaniya were better motivated towards osteoporosis preventive behaviour. These differences cannot be directly explained based on the data available from this study.

Among the limitations of the study is the fact that only two medical schools were included. Also there might be other contributing factors that were not explored in this study, such as socio-economic differences, access to facilities such as access to sports equipment, gymnasiums, swimming pools and nutritious meals although the study provides baseline data for further study. These factors might be contributing towards the differences seen in health motivation and practices. Future research specifically targeted towards socio-economic factors, access to facilities and health beliefs will be useful in understanding these differences. Also future qualitative studies may be helpful in exploring the reasons for these differences.

This study highlights the need for health education targeted to young females on osteoporosis, improving calcium intake and physical activity. In addition, primary health care interventions such as preventive health education may help to reduce the burden of osteoporosis in the community.

## Conclusions

Although majority of participants had a modest knowledge on osteoporosis, there were important gaps in knowledge in relation to risk factors and protective factors of osteoporosis and on the insidious nature of osteoporosis. Perceived susceptibility for osteoporosis was low. Practices towards preventing osteoporosis were inadequate. This study indicates a lack of knowledge on osteoporosis in young female undergraduates in Sri Lanka and highlights the need for health education on osteoporosis targeted to young females.

## Abbreviations

OKAT: Osteoporosis knowledge assessment tool
FAO: Food and agriculture organisation
WHO: World Health Organisation
RDA: Recommended daily allowances
BMD: Bone mineral density

## References

[1] *International Osteoporosis Foundation (IOF)*. [http://www.osteofound.org/facts-statistics]

[2] Mithal A, Dhingra V, Lau E: *The Asian Audit: Epidemiology, Costs and Burden of Osteoporosis in Asia*. International Osteoporosis Foundation 2009; 24–9. [http://www.iofbonehealth.org/sites/default/files/PDFs/Audit%20Asia/Asian_regional_audit_2009.pdf]

[3] Lau EM (2009). The epidemiology of osteoporosis in Asia. IBMS Bone Key.

[4] Juby AG, Davis P (2001). A prospective evaluation of the awareness, knowledge, risk factors and current treatment of osteoporosis in a cohort of elderly subjects. Osteoporos Int.

[5] Yin-King Lee L, King-Fai Lai E (2006). Osteoporosis in older Chinese men: knowledge and health beliefs. J Clin Nurs.

[6] Terrio K, Auld GN (2002). Osteoporosis knowledge, calcium intake, and weight-bearing physical activity in three age groups of women. J Community Health.

[7] Pande K, Pande S, Tripathi S, Kanoi R, Thakur A, Patle S (2005). Poor knowledge about osteoporosis in learned Indian women. J Assoc Physicians India.

[8] Eyigör S, Karapolat H, Durmaz B (2008). Medical Students’ knowledge of osteoporosis in Ege University Faculty of Medicine. Rheumatism.

[9] Riaz M, Abid N, Patel J, Tariq M, Khan MS, Zuberi L (2008). Knowledge about Osteoporosis among healthy women attending a tertiary care hospital. J Pak Med Assoc.

[10] Liew YL, Mann D, Piterman L (2002). Osteoporosis risks. A comparative study of Asian Australian and Caucasian Australian women. Aust Fam Physician.

[11] Schwab P, Klein RF: **Non pharmacological approaches to improve bone health and reduce osteoporosis.** *Curr Opin Rheumatol* 2008, **20**. [http://journals.lww.com/co-rheumatology/pages/articleviewer.aspx?year=2008&issue=03000&article=00017&type=abstract] 10.1097/BOR.0b013e3282f3cbd3 18349754

[12] Mazess RB, Barden HS (1991). Bone density in pre-menopausal women: effects of age, dietary intake, physical activity, smoking, and birth-control pills. Am J Clin Nutr.

[13] Hannan MT, Felson DT, Dawson-Hughes B, Tucker KL, Cupples LA, Wilson PW, Kiel DP (2000). Risk factors for longitudinal bone loss in elderly men and women: the Framingham osteoporosis study. J Bone Miner Res.

[14] Kasper MJ, Peterson MGE, Allegrante JP, Galsworthy TD, Gutin B (1994). Knowledge, beliefs and behaviours among college women concerning the prevention of osteoporosis. Arch Fam Med.

[15] Winzenberg TM, Oldenburg B, Frendin S, Jones G (2003). The design of a valid and reliable questionnaire to measure osteoporosis knowledge in women: the Osteoporosis Knowledge Assessment Tool (OKAT). BMC Musculoskelet Disord.

[16] Kim KK, Horan ML, Gendler P, Patel MK (1991). Development and evaluation of the osteoporosis health belief scale. Res Nurs Health.

[17] Andrea HT, Robertson TP, Sellmeyer DE (2009). Validation of two food frequency questionnaires for dietary calcium assessment. J Am Diet Assoc.

[18] Taylor C, Lamparello B, Kruczek K, Anderson EJ, Hubbard J, Misra M (2009). Validation of a food frequency questionnaire for calcium and vitamin D intake in adolescent girls with anorexia nervosa. J Am Diet Assoc.

[19] Medical Research Institute: *Recommended Dietary Allowances for Sri Lankans*. Department of Nutrition, Medical Research Institute; 2007.

[20] Hernandez-Rauda R, Martinez-Garcia S (2004). Osteoporosis-related life habits and knowledge about osteoporosis among women in El Salvador: a cross-sectional study. BMC Musculoskelet Disord.

[21] Ribeiro V, Blakeley J, Laryea M (2000). Women’s knowledge and practices regarding the prevention and treatment of osteoporosis. Health Care Women Int.

[22] Von Hurst PR, Wham CA (2007). Attitudes and knowledge about osteoporosis risk prevention: a survey of New Zealand women. Public Health Nutr.

[23] Edmonds E, Turner LW, Usdan SL (2012). Osteoporosis knowledge, beliefs, and calcium intake of college students: utilization of the health belief model. Open J Prev Med.

[24] Farr JN, Blew RM, Lee VR, Lohman TG, Going SB (2011). Associations of physical activity duration, frequency, and load with volumetric BMD, geometry, and bone strength in young girls. Osteoporos Int.

[25] Bleicher K, Cumming RG, Naganathan V, Seibel MJ, Sambrook PN, Blyth FM, Le Couteur DG, Handelsman DJ, Creasey HM, Waite LM (2011). Lifestyle factors, medications, and disease influence bone mineral density in older men: findings from the CHAMP study. Osteoporos Int.

[26] Wallace LS, Ballard JE (2002). Lifetime physical activity and calcium intake related to bone density in young women. J Womens Health Gend Based Med.

[27] Hu JF, Zhao XH, Jia JB, Parpia B, Campbell TC (1993). Dietary calcium and bone density among middle-aged and elderly women in China. Am J Clin Nutr.

[28] Ho SC, Leung PC, Swaminathan R, Chan C, Chan SSG, Fan YK, Lindsay R (1994). Determinants of bone mass in Chinese women aged 21–40 years. II. Pattern of dietary calcium intake and association with bone mineral density. Osteoporos Int.

[29] Fleming KH, Heimbach JT (1994). Consumption of calcium in the US; food sources and intake levels. J Nutr.

